# Primary Central Nervous System (CNS) Post-Transplant Lymphoproliferative Disorder: A Case Report

**DOI:** 10.7759/cureus.95108

**Published:** 2025-10-21

**Authors:** Jared M Taitt, Christopher Lees, George N Tran, Satya Patel

**Affiliations:** 1 Internal Medicine, University of California Los Angeles David Geffen School of Medicine, Los Angeles, USA; 2 Internal Medicine, Veterans Affairs Greater Los Angeles Healthcare System, Los Angeles, USA

**Keywords:** ebv ptld, immunosuppression complications, post-renal transplant complications, post transplant lymphoproliferative disorder, rare cause of altered mental status

## Abstract

Post-transplant lymphoproliferative disorder (PTLD) is the most common non-dermatologic malignancy in patients with a history of solid organ transplantation (SOT). The onset of PTLD is not limited to a specific timeframe post-SOT, but most cases are diagnosed within five years of transplantation. While both metastatic spread and multifocal involvement are common, very few of these cases arise from the central nervous system. Here, we present a patient with progressive memory loss 15 years after renal transplantation who was found to have primary central nervous system PTLD. This case underscores the importance of continued vigilance in post-transplantation care.

## Introduction

Post-transplant lymphoproliferative disorder (PTLD) is a well-recognized complication in patients receiving long-term immunosuppression following solid organ transplantation (SOT), with the majority of cases associated with Epstein-Barr virus (EBV) positivity. Following acute infection, the virus persists in memory B cells by downregulating antigen presentation, thereby allowing for immune evasion. In immunocompetent individuals, T-cell surveillance suppresses reactivation. However, in SOT patients receiving T cell-targeting immunosuppression, this mechanism of defense is hindered, resulting in reactivation of EBV and transformation of B cells into immortalized lymphoblastoid cells [1]. As such, this iatrogenic immunocompromised state lies the foundation for the development of lymphoproliferative disorders.

PTLD can manifest both within and outside the lymphatic system. While extranodal disease is common, central nervous system (CNS) involvement is rare, particularly as the sole focus of disease. As such, primary central nervous system post-transplant lymphoproliferative disorders (PCNS-PTLD) represent an exceedingly rare and complex subset of PTLD, comprising between 5 and 30%, with poorly understood risk factors and disease course given the paucity of cases [2,3]. Of observed cases of PCNS-PTLD, 79% were diagnosed in patients who had undergone renal transplantation, 30% were in patients who had received their transplants greater than 10 years prior to diagnosis, and 88% were found to be positive for EBV on histology [4]. Herein, we present a case of PCNS-PTLD in a 64-year-old male 15 years after renal transplantation.

## Case presentation

**Figure 1:**
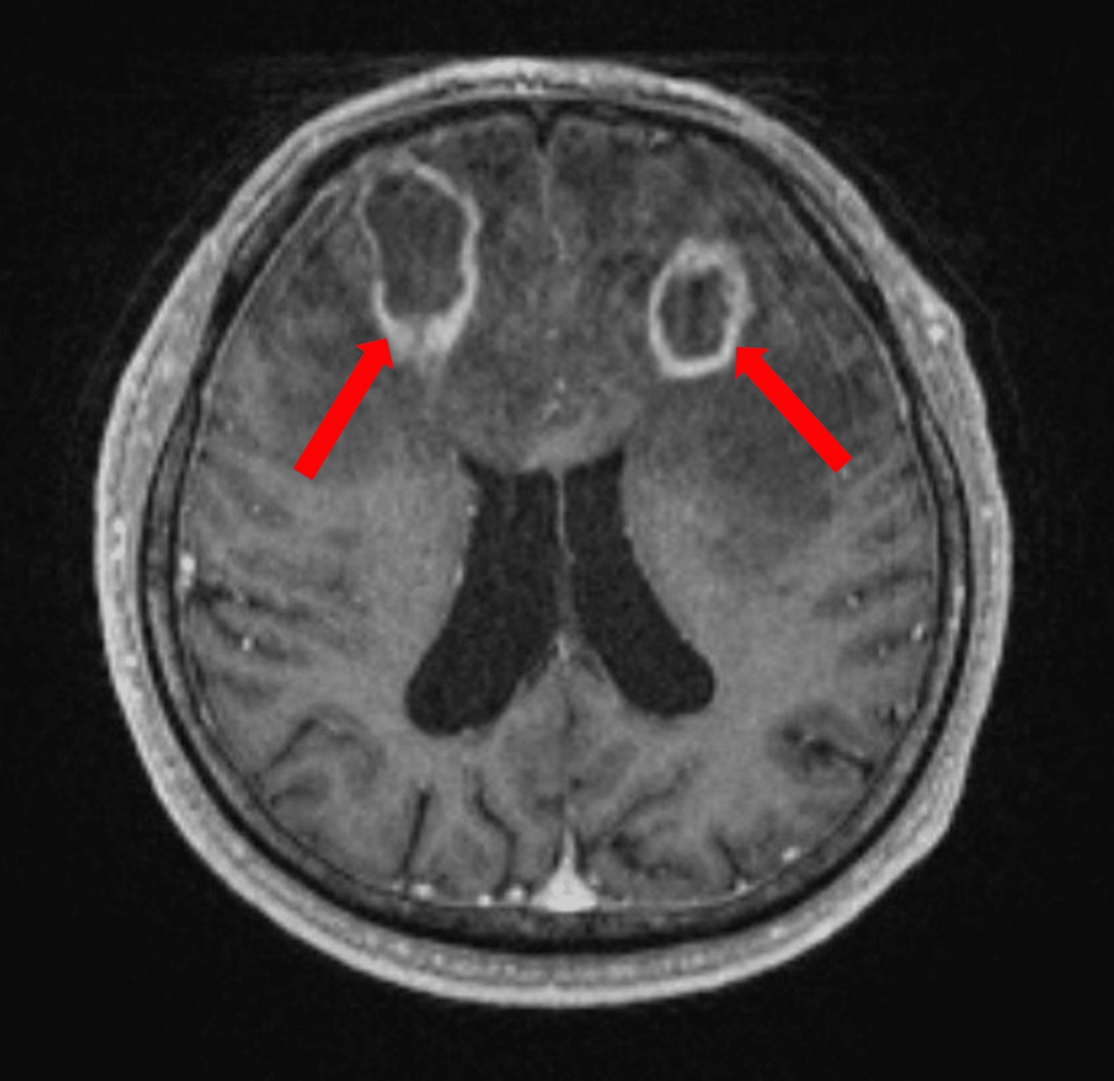
MRI of the brain without contrast Notable for two prominent peripheral rim-enhancing and diffusion-restricting lesions associated with extensive surrounding edema MRI: Magnetic resonance imaging

A 64-year-old male with a history of renal transplantation to the right retroperitoneum 15 years prior to admission presented to the Emergency Department with progressive forgetfulness for several months. His symptoms acutely worsened during the preceding week, prompting him to seek medical evaluation. He was on a long-term immunosuppression regimen including cyclosporine and mycophenolate mofetil. The patient’s vitals were significant for a blood pressure of 214/93 mm Hg. He was otherwise afebrile and had no focal infectious symptoms. A physical exam revealed a mild right-sided ptosis of unclear chronicity and psychomotor slowing on interview. Laboratory testing was notable for leukopenia with a white blood cell count of 3.88 x103 cells/uL (normal: 4.16-9.95 x103 cells/uL) and an elevated lactate dehydrogenase of 382 U/L (normal: 125-256 U/L). Given his memory loss and focal neurological symptoms, a non-contrast computed tomography (CT) of his head was ordered, revealing multiple peripheral ring-enhancing lesions (Figure 1). While these findings were initially concerning for metastatic cancer of unknown primary site, CT imaging of the chest, abdomen, and pelvis was negative for a primary lesion. An extensive infectious workup, including serologic testing for HIV, EBV, toxoplasmosis, and syphilis, was negative. A lumbar puncture was performed, and cerebrospinal fluid showed an elevated protein of 162 mg/dL (normal: 15-45 mg/dL), elevated glucose of 114 mg/dL (normal: 43-73 mg/dL), elevated white blood cells of 84 cells/cmm (normal: 0-5 cells/cmm) that were 86% lymphocytes (normal: 40-80%), and an unremarkable Gram stain. Cytology and flow cytometry of the cerebrospinal fluid were normal. Ultimately, a brain biopsy was performed, which revealed EBV-positive monomorphic PTLD, diffuse large B-cell lymphoma (DLBCL) type.

After the diagnosis had been established, a positron emission tomography-computed tomography scan was performed to assess systemic involvement. However, there was no evidence of fluorodeoxyglucose uptake other than previously visualized bilateral cortical lesions. The patient was subsequently started on the TEDDI-R (temozolomide, etoposide, doxil, dexamethasone, ibrutinib, rituximab) treatment regimen [5]. According to the standard of care for PTLD, the patient’s cyclosporine dose was also decreased. The patient received two doses of rituximab and one cycle of the remaining TEDDI-R agents; however, his treatment course was discontinued after he developed acute kidney injury and subsequent renal failure requiring hemodialysis. Follow-up brain magnetic resonance imaging with contrast at six weeks post-TEDDI-R showed an increased non-enhancing mass-like T2/FLAIR signal in the right frontal lobe, an increased size of a left precuneus lesion, but otherwise an interval decrease in size of the remaining rim-enhancing lesions, likely representing a mixed response to treatment. His clinical course was further complicated by pancytopenia, neutropenia, and septic shock secondary to typhlitis. He later developed a lower gastrointestinal bleed secondary to tissue-invasive cytomegalovirus colitis. The patient, unfortunately, succumbed to his illness and died two months after his initial diagnosis.

## Discussion

Advances in post-transplantation care over recent decades have significantly extended survival in SOT recipients [6]. As a result, long-term complications once considered rare are gaining increased clinical relevance. This shift highlights the importance of reexamining the epidemiology, pathogenesis, and diagnostic approach of PCNS-PTLD, which warrants review. Further, clinicians should also consider a broader differential in patients with a remote history of SOT who present with vague neuropsychiatric complaints, much as they would when assessing unexplained fever in this population.

PCNS-PTLD and PTLD with secondary CNS lesions may present with a diverse array of symptoms, including headaches, seizures, altered mental status, and focal neurologic deficits [2]. The mean time to diagnosis after SOT is 4.5 years; however, this can vary depending on individual patient characteristics and the immunosuppressive regimen with which the patient is treated [3]. Risk factors for earlier-onset PCNS-PTLD (within one year of SOT) include patients who are young, have EBV-negative status prior to transplantation, have multi-organ transplantations, and have allograft involvement. Risk factors for later-onset PCNS-PTLD are older age, a history of hepatitis C infection, and the use of calcineurin inhibitors such as cyclosporine and tacrolimus [2]. Our patient’s presentation, occurring 15 years post-transplantation at the age of 64 years old and involving cyclosporine and mycophenolate use, aligns with known risk factors for late-onset disease. Mycophenolate mofetil also represents a potential risk factor for patients transitioned to this medication from other immunosuppressive regimens [7] and patients started on this medication for autoimmune comorbidities [8]. On neuroimaging, PCNS-PTLD lesions tend to present as lobar predominant, ring-enhancing with ill-defined margins, CT hypoattenuation, MRI T1 hypointensity, and T2/FLAIR hyperintensity [2,9].

Even in the presence of clinical, radiographic, and demographic features, diagnosing PCNS-PTLD remains challenging. Many of these features often overlap with the sequelae of other infectious, malignant, and vascular processes [9]. In the context of immunosuppression, the differential is further broadened to include atypical and opportunistic infections. Studies evaluating the efficacy of serological markers such as EBV-DNA derived from serum and cerebrospinal fluid alike have shown limited diagnostic utility [10]. Consequently, after less invasive tests are done to rule out the more common disease processes with a similar presentation, a definitive diagnosis of PCNS-PTLD often requires a stereotactic biopsy from a CNS lesion [11].

Histopathologic diagnosis of PCNS-PTLD on pathology incorporates morphologic, immunophenotypic, and genetic features. The clonality, presence of EBV-encoded RNA (EBER), cytology, and the characteristics of lymphoid architecture further allow for the classification of PTLD subtypes. As in our case, effacement of normal tissue architecture by sheets of monoclonal malignant B-cells is seen in DLBCL-type PTLD, which represents the most common subtype of monomorphic PTLD [12]. These B-cells often contain large nuclei, multiple peripheral nucleoli, and a narrow rim of basophilic cytoplasm; express pan-B-cell antigens; and almost universally stain positive for EBER [13].

While there are a multitude of factors that inform management for PCNS-PTLD, including the type of organ transplanted, histological subtype, and institutional guidelines [14], two goals remain constant: treatment of the malignancy and preservation of graft function. For PCNS-PTLD, initial therapy often includes the reduction of the immunosuppressive medication with the aim of increasing T-cell-mediated destruction of EBV-infected malignant cells, though few cases resolve through this intervention alone [15].

Careful consideration must be taken when determining the extent of reduction of immunosuppression, including the type of organ transplanted and the potential risk of graft rejection and failure. In conjunction with this approach, a mainstay of therapy for monomorphic CD20-positive PTLD is rituximab [16,17]. Single-agent treatment with rituximab only achieves complete remission in approximately 20% of cases and often requires treatment in conjunction with a chemotherapeutic regimen. Among the most widely studied regimens for patients with CD20-positive PTLD is R-CHOP, which adds cyclophosphamide, doxorubicin, vincristine, and prednisone [18]. A similar regimen, CHOP, which excludes rituximab, is used for CD20-negative cases of PTLD. Recently, single-agent ibrutinib [19] as well as TEDDI-R [20] have demonstrated efficacy in the treatment of PCNS-PTLD in limited trials and warrant further investigation for potential superior benefit in treating this disease.

## Conclusions

As the long-term outcomes of SOT recipients continue to improve, it is vital to recognize rare pathologies that may be increasing in incidence due to an evolving clinical macroenvironment. In patients presenting post-SOT with focal neurologic and/or cognitive symptoms, as well as imaging remarkable for intracranial lesions without extracranial organ involvement, PCNS-PTLD warrants consideration. This diagnosis requires the incorporation of clinical context, neuroimaging, and histology, given an often variable clinical presentation. While optimal therapies remain under investigation, emerging evidence suggests a benefit in the treatment with TEDDI-R and warrants further investigation.
